# To Canvassers

**Published:** 1885-12

**Authors:** 


### TO CANVASSERS.

Having come to the conclusion to push the circulation of the Journal up to one hundred thousand as soon as it can be done, we want, in every township, village and city, in the United States, Canada and Australia, agents who will take hold of the business as if they were in earnest. We will give good agents a chance to make money. Let them address enquiries to this office.

In Malabar a tree called the tallow tree grows; from the seeds of it, when boiled, is procured a firm tallow which makes excellent candles.

If hens are to be kept in Winter with a view to profit, they must have comfortable accommodations, where they can be protected from extreme cold, and have a southern or southeastern exposure, where the sunlight can be admitted.

Durability of Woods.—In some tests made with small squares of various woods buried an inch in the ground, the following results, says the *Garden*, were noted: Birch and aspen decayed in 3 years; willow and horse chestnut in 4 years; maple and red beech in 5 years; elm, ash, hornbeam and Lombardy poplar in 7 years; oak, Scotch fir, Weymouth pine and silver fir decayed to a depth of half an inch in 7 years; larch, juniper and arbor-vitæ were uninjured at the expiration of the 7 years.

Flaxseed.—The receipts of flaxseed in Chicago during the last month reached the astounding aggregate of 1,875,000 bushels of 56 pounds each, and during one week the receipts of the seed actually exceeded those of wheat. For the first three months of the current year the receipts were nearly 1,600,000 bushels larger than, and the shipments were more than 1,500,000 bushels in excess of those for the corresponding time last year. About 250,000 bushels have been already exported, and as much more is under orders to move across the ocean. It will take the place in the old world of so much Calcutta seed, and perhaps make amends for an alleged failure of the crop in Russia.

Catching Cold.—I made a discovery that has proved invaluable to me, and I believe also to others to whom it has been imparted; and that has made catarrh, to me, very much less formidable than it used to be. It is, that all the most violent symptoms, the running at eyes and nose, sneezing, lassitude, weariness, and the thousand and one distressing feelings that are included in the equipment of this well-known complaint, may be completely suppressed, simply by abstaining from all liquid food and drink of every kind for a period varying in my own case, from 24 to 50 hours, according to the violence of the symptoms. Take all the food dry, drink nothing, not even a cup of tea nor a spoonful of soup.

